# s-Block metallabenzene: aromaticity and hydrogen adsorption

**DOI:** 10.1007/s00894-014-2552-6

**Published:** 2015-01-29

**Authors:** Rafał Roszak, Szczepan Roszak

**Affiliations:** Institute of Physical and Theoretical Chemistry, Wroclaw University of Technology, Wybrzeze Wyspianskiego 27, 50-370 Wroclaw, Poland

**Keywords:** Metallabenzene, Metallaromaticity, Kubas interactions, Hydrogen adsorption, Hydrogen storage

## Abstract

Second group metal dimers can replace the carbon atom in benzene to form metallabenzene (C_5_H_6_M_2_) compounds. These complexes possess some aromatic character and promising hydrogen adsorption properties. In this study, we investigated the aromatic character of these compounds using aromaticity indices and molecular orbital analysis. To determine the nature of interactions between hydrogen and the metallic center, variation-perturbational decomposition of interaction energy was applied together with ETS-NOCV analysis. The results obtained suggest that the aromatic character comes from three π orbitals located mainly on the C_5_H_5_^−^ fragment. The high hydrogen adsorption energy (up to 6.5 kcal mol^−1^) results from two types of interaction. In C_5_H_6_Be_2_, adsorption is controlled by interactions between the empty metal orbital and the σ orbital of the hydrogen molecule (Kubas interaction) together with corresponding back-donation interactions. Other C_5_H_6_M_2_ compounds adsorb H_2_ due to Kubas interactions enhanced by H_2_–π interactions.

**Electronic supplementary material:**

The online version of this article (doi:10.1007/s00894-014-2552-6) contains supplementary material, which is available to authorized users.

**Graphical Abstract:**
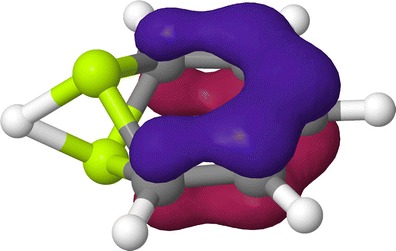
First π orbital in C_5_H_6_Be_2_

## Introduction

The concept of aromaticity was defined in the nineteenth century to explain the unusual properties of benzene. Nowadays, this term is also used to describe other compounds including heterocyclic [1] and metallacyclic compounds [2–4] as well as metal clusters [5, 6]. Since the term “aromaticity” is used for many different types of resonance stabilization phenomena, the classification of compounds as aromatic or non-aromatic is not always obvious. The quantitative determination of this property is possible via introduced aromaticity indices (AI). Several AI have been proposed; however, all of them are based on one of the following ring criteria: geometrical, energetic, magnetic or electronic properties. The geometrical aspect of aromaticity is measured predominantly by the HOMA (harmonic oscillator model of aromaticity) index proposed by Kruszewski and Krygowski [7]. HOMA is the most commonly used AI; however, this index depends on empirical parameters that are defined for organic compounds only. Therefore HOMA cannot be applied to organometallic compounds. NICS (nucleus-independent chemical shift) represents a series of indices [8] that allow evaluation of aromaticity on the basis of the magnetic properties of the ring. NICS uses a negative value of absolute shielding in the given position. NICS indices do not depend on empirical parameters and thus may be applied easily to any aromatic system. A negative value of an index indicates that the compound is aromatic whereas positive values states that the investigated compound is antiaromatic. There are three commonly used NICS indices: NICS(0), defined as negative value of NICS in the ring center; NICS(1), which represents negative value of NICS measured 1 Å above the center of a ring; and NICS(1)zz, which corresponds to anisotropic part of NICS(1) in the direction perpendicular to the ring. The ring center is usually defined as a geometric center; however, it can also be defined as a ring critical point (RCP) based on the atoms in molecules (AIM) theory introduced by Bader [9].

The key feature of aromatic compounds is a delocalization of π electrons, which leads to additional stabilization of a structure. The stabilization energy is called the resonance energy or aromatic stabilization energy (ASE). In general, ASE represents the energy difference between an aromatic compound and its non-aromatic unsaturated isomer. ASE can be calculated in the isodesmic reaction [10], where substrates and products have the same number of atoms, bonds, and bond types. The standard example constitutes the hypothetical reaction of three cyclohexenes that form benzene and two cyclohexanes. The other reaction suited to calculating ASE is a double-bond migration isomerization reaction [11]. In this reaction, the product constitutes an aromatic compound with a methyl substituent in the meta position whereas the substrate has a methylidene substituent and an additional hydrogen in the para position.

A number of indices are based on electronic properties. The aromaticity of a molecule can be characterized by properties of electron density calculated in RCP. Palusiak et al. [12] and Ebrahimi et al. [13] proved that the value of electron density, laplacian of electron density, and energies of electron density, including potential (V), kinetic (G) and total (H) electron energy, are strongly correlated with a HOMA value. Besides electronic properties in bond critical point (BCP), several other indices are defined within the AIM framework. However, most common indexes [14], e.g., PDI (para-delocalization index) [15], ΔDI [15], FLU [16] and FLU_π_ [16], cannot be applied to C_5_M_2_H_6_ compounds. The reasons are as follows [14]: by definition, the PDI index can be used only for 6-membered rings; ΔDI requires a clearly defined Lewis structure; FLU, like HOMA, needs empirical parameters for each bond type whereas FLU_π_ can be used only for planar compounds. One of few AIM-based indices that can be applied in the studied case is the Shannon aromaticity (SA) index [17] based on the Shannon entropy [18]. The formula describing SA is presented below:

1 $$ SA= \ln (N)-{\displaystyle \sum_i^N}{p}_i \ln {p}_i $$

where

2 $$ {p}_i=\frac{\rho_{BCPi}}{{\displaystyle \sum_{j=1}^N{\rho}_{BCPj}}} $$

with N representing the overall number of bonds in the ring and ρ_BCP_ being electron density in the BCP. The value 0 corresponds to the homonuclear aromatic ring. SA increases when aromaticity decreases.

Several metalloaromatic compounds have been reported in literature and the topic is covered by several reviews [2–4]. Most of these compounds are benzene analogues where CH groups are replaced by transition metals with ligands, so called metallabenzenes [19, 20]. Compounds of this type containing transition metal atoms have been obtained experimentally and reveal aromatic properties (e.g., undergo aromatic substitution). Fernandez and Frenking [21] have proved that metallabenzenes containing Os, Ru, Ir, Rh, Pt, and Pd possess five π orbitals and can be considered as 10 electron Hückel aromatic systems.

Recently, we reported that the Be_2_ fragment, which is isoelectronic with the carbon atom, can act as C(sp^2^) [22]. The Be_2_ center can replace the carbon atom in aromatic hydrocarbons and form compounds characterized by structure and molecular orbitals analogous to that in corresponding hydrocarbons. The aim of this study was to characterize the bonding scheme and aromaticity of compounds hosting Be_2_ and other M_2_ fragments (with M being second group metals; Table 1).

**Table 1:** Aromaticity of C_5_H_6_M_2_ [electron density properties at ring critical point (RCP)]. *NBO* Natural bond orbital, *NICS* nucleus-independent chemical shift, *SA* Shannon aromaticity, *ASE* aromatic stabilization energy, *H* total electron energy, *G* kinetic electron energy, *V* potential electron energy

Compound^a^	C_5_H_6_Be_2_	C_5_H_6_Mg_2_	C_5_H_6_Ca_2_	C_5_H_6_BeMg	C_5_H_6_BeCa	C_5_H_6_MgCa
NBO charge (e)	Be: 0.84	Mg: 1.162	Ca_up_: 1.327	Be: 0.540	Be: 0.573	Mg: 1.110
			Ca_down_: 1.311	Mg: 1.220	Ca: 1.557	Ca: 1.367
NICS(O)	1.42	−0.83	1.66	0.12	0.01	−0.72
NICS(O)_zz_	1.60	−5.30	0.48	−6.71	2.61	−1.39
NICS(1)	−3.63	−4.42	−0.49	−3.83	−3.41	−3.45
NICS(1)_ZZ_	−16.68	−16.23	−3.14	−18.05	−4.80	−10.04
Electron density	0.0181	0.0134	0.0113	0.0166	0.0168	0.0124
Laplacian	0.1095	0.0661	0.0457	0.0939	0.0919	0.0538
H	0.0049	0.0028	0.0015	0.0042	0.0040	0.0020
G	0.0225	0.0137	0.0099	0.0193	0.0190	0.0115
V	−0.0176	−0.0108	−0.0083	−0.0151	−0.0150	−0.0095
SA *10^2^	9.809^C^	15.026^C^	20.404^Cadown^	12.355^Be^	24.782^Be^	19.927^Mg^
	13.619^Be^	21.383^Mg^		22.854^Mg^		
ASE (kcal mol^−1^)	3.6	7.7	18.6	24.8	35.6	33.6

^a^C: all C ring, Be: ring with 5 C and Be, Mg: ring with 5 C and Mg, Ca_down_: ring with 5 C and Ca_down_

Compounds including a Be_2_ center constitute promising materials for hydrogen storage, and can adsorb hydrogen with energy up to 6 kcal mol^−1^. Hence, the second aim of this work was to characterize interactions of the M_2_ moiety with hydrogen molecule(s). Here, we are concentrating on metallabenzene (C_5_M_2_H_6_). Previous studies on Be_2_-containing compounds have shown that C_5_Be_2_H_6_ preserves all features of higher aromatic hydrocarbons.

## Computational details

If not mentioned otherwise, molecular structures and complexes with hydrogen molecule(s) were minimized at MP2 level of approximation [23] applying the aug-cc-pvdz atomic basis set [24]. Harmonic vibration frequencies revealed no imaginary frequencies indicating that structures correspond to true minima. NICS values were calculated using the gauge-independent atomic orbital (GIAO) method [25] applied at the same level of theory. Calculations mentioned above were performed in the Gaussian 09 software suite [26].

In order to investigate the nature of interactions between the M_2_ center and molecular hydrogen, the total MP2 interaction energy was decomposed according to the hybrid variational-perturbational scheme [27]. In adopted scheme the total interaction energy is partitioned into Hartree-Fock (HF) (Δ*E*_HF_) and second order perturbation (*E*_MP2_) terms:

3 $$ \varDelta {\mathrm{E}}_{\mathrm{MP}2} = \varDelta {\mathrm{E}}_{\mathrm{HF}} + {\mathrm{E}}_{\mathrm{MP}2} = {\mathrm{E}}_{\mathrm{el}} + {\mathrm{E}}_{\mathrm{del}} + {\mathrm{E}}_{\mathrm{ex}} + {\mathrm{E}}_{\mathrm{MP}2} $$

The HF interaction energy is further decomposed into first order electrostatic (*E*_el_), Heitler-London exchange (*E*_ex_) and higher order delocalization (*E*_del_) terms. All energy contributions are calculated in the dimer-centered basis set and hence are free from basis set superposition error (BSSE). The interaction energy calculations were performed utilizing the GAMESS software [28] incorporating EDS modifications [29].

The interaction energy was also calculated applying the ETS-NOCV scheme [30] based on the ETS protocol [31]. In the ETS method, the total interaction energy (Δ*E*_int_) consists of contributions from orbital interactions (*E*_orb_), Pauli repulsion (*E*_pauli_) and electrostatic interaction (*E*_elst_):

4 $$ \varDelta {\mathrm{E}}_{\mathrm{int}} = {\mathrm{E}}_{\mathrm{orb}} + {\mathrm{E}}_{\mathrm{pauli}} + {\mathrm{E}}_{\mathrm{elst}} $$

The NOCV approach [32] makes it possible to decompose deformation electron density into a set of NOCV density-differential pairs (charge flow channels) and to estimate the contribution of each pair to the orbital energy (*E*_orb_). The ETS-NOCV decomposition was calculated based on B3LYP functional and ADZVP basis set [33] build from Slater functions using ADF software [34–36].

In both decomposition schemes, electrostatic and Pauli exchange terms describe the same interactions, namely, electrostatic attraction and Pauli repulsion between unperturbed electron density of monomers. *E*_orb_, *E*_ex_ and *E*_MP2_ terms describe the energetic effect of electron density change between separate monomers and the complex. The ETS-NOCV scheme is implemented for DFT methods only and obtained energies are not corrected for basis-set superposition error. Therefore we use the hybrid variational-perturbational scheme for the decomposition and ETS-NOCV for further characterization of orbital interactions only. Energy values in both decomposition schemes are not equal due to the different level of theory and basis sets type (Gaussian vs Slater function) but are in close agreement (typically below 1 kcal mol^−1^), thus allowing the qualitative discussion of orbital interactions.

Contours of molecular orbitals (MO) were plotted using the Jmol program [37] whereas NOCV charge flow channels were visualized using ADF-GUI software [38]. Contour values were adjusted individually to provide optimal readability of the figures (values 0.05–0.07 a.u. where used for MOs, and 0.002–0.005 for charge flow channels). AIM analysis was performed using the MultiWfn program [39].

## Results and discussion

### “Be_2_-benzene”

A beryllium dimer incorporated into carbon networks can be treated as a superatom. The Be_2_ fragment is isoelectronic with the single carbon atom and its orbitals can mimic C(sp^2^) hybridization [22]. This approach suggests that the Be_2_ fragment should be involved in a π electron delocalization in C_5_Be_2_H_6_. On the other hand, “Be_2_-benzene” can be treated as a coordination compound with the C_5_H_5_^−^ ligand. In such a situation, the π delocalization (and aromaticity) depends on the ligand and does not involve the Be_2_ center. The second hypothesis is based on the work of Fernandez and Frenking [21], which states that [(C_5_H_5_)Rh(PH_3_)_2_(Cl_2_)] can be treated as a coordination compound with C_5_H_5_^−^ ligand. Molecular orbitals of “Be_2_-benzene” carring π electrons (Table 4) are similar to these in silabenzene and germabenzene (Table S3). The first MO possesses only a single nodal plane and spans over all the carbon atoms. The remaining two MOs possess two nodal planes; however, in contrast to C_5_H_6_Si and C_5_H_6_Ge, the last π MO in “Be_2_-benzene” shows significant density depletion on the heteroatomic center. The AIM analysis revealed (Table S5) that each beryllium atom is involved in bonding interactions with both neighboring carbons (denoted as C_orto_) and nearest hydrogen atoms. The BCP was also found on the path connecting both C_orto_ atoms. The laplacian in all BCPs is positive, indicating closed shell (ionic) interactions. The results of AIM analysis suggest that “Be_2_-benzene” has a partially ionic structure and can be considered as a coordinating compound with C_5_H_5_^−^ and H^−^ ligands around the Be_2_^2+^ center. Aromatic indices cannot give a clear answer about the aromaticity of “Be_2_-benzene”. NICS(0) shows no aromaticity whereas NICS(1) (and especially NICS(1)zz) suggests significant aromatic character. Based on magnetic properties, the “Be_2_-benzene” molecule is less aromatic compared to the C_5_H_5_^−^ ligand alone (Table S1). Electron density properties in RCP and SA index state that “Be_2_-benzene” is the most aromatic compound of the C_5_H_6_M_2_ series. The opposite conclusion can be drawn from ASE, which suggests that C_5_H_6_Be_2_ is the least aromatic of the series.

The Be_2_ fragment can adsorb one hydrogen molecule with relatively high energy (5.1 kcal mol^−1^). The adsorption of first hydrogen is controlled by the Kubas-like interaction. The classical Kubas interaction [40] involves the σ-orbital of the hydrogen molecule and the empty d-orbital of the metal. Beryllium does not possess any d orbitals; instead, a p-orbital is involved in interactions. In the ETS-NOCV analysis, in addition to the Kubas-like interaction based on electron transfer from hydrogen to metal, a strong back-donation is observed. The adsorption of a second hydrogen on Be_2_ due to lower energy of the Kubas-like interaction is relatively low (1.9 kcal mol^−1^).

### “Mg_2_-benzene”

The geometry (Table 2) and molecular orbitals (Tables 3, 4) of C_5_H_6_Mg_2_ (“Mg_2_-benzene”) are similar to those observed in “Be_2_-benzene”. AIM analysis indicates the same bonding scheme. Hence, the compound can be also treated as a complex with an aromatic C_5_H_5_^−^ ligand. The aromaticity of “Mg_2_-benzene” based on magnetic properties calculated 1 Å above the ring is comparable to the “Be_2_-benzene” case. In contrast to “Be_2_-benzene”, NICS values for “Mg_2_-benzene” in RCP are negative, confirming the aromatic character of the compound. The electron density properties suggest lower aromaticity, whereas ASE suggests higher aromaticity in comparison to “Be_2_-benzene”.

**Table 2:**
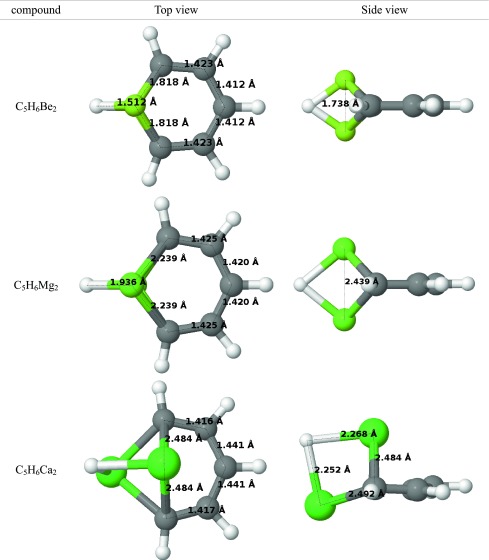

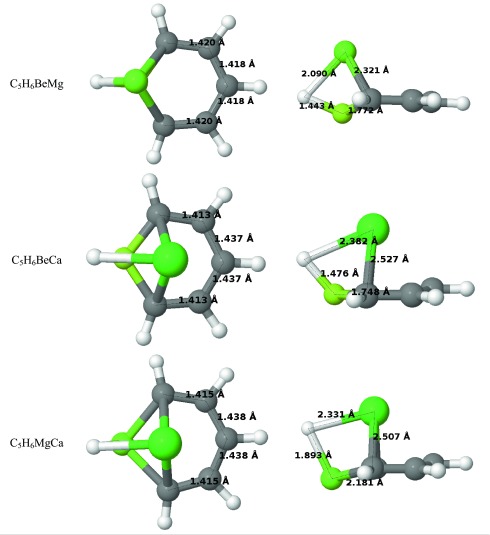
Optimized structures of C_5_H_6_M_2_

**Table 3:**
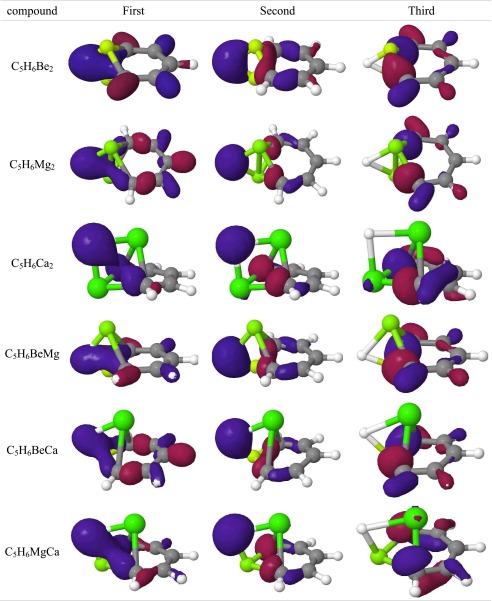
Molecular orbitals (MO) involved in bonding of metal atoms. The orbitals are ordered by increasing energy (the first has the lowest energy)

**Table 4:**
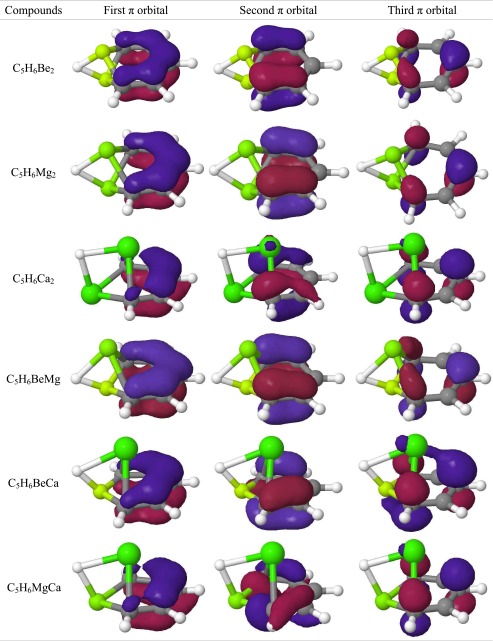
π-Type MOs in C_5_H_6_M_2._ The orbitals are ordered by increasing energy (the first has the lowest energy)

The adsorption energy of first hydrogen molecule on “Mg_2_-benzene” is lower than that of “Be_2_-benzene” (3.8 kcal mol^−1^). The adsorption of this molecule is controlled by electrostatic and delocalization terms that constitute almost equal contribution and are two times higher than correlation effects. The geometry of the complex shows that the distances between magnesium and hydrogen atoms from adsorbed H_2_ molecule are not equal. The first hydrogen atom (denoted as H1) is attached directly to Mg whereas the second (denoted as H2) is in contact with the Mg and C_para_ atoms. This may suggest that the hydrogen molecule interacts via the empty orbital on magnesium and also with π-orbitals located on carbons. ETS-NOCV reveals that three deformation density channels are responsible for 94 % of the orbital energy (Table 5). The first charge flow channel, which is responsible for 60 % of orbital interactions, indicates an increase in electron density on H1 and between magnesium and the H1 atom. The same charge flow channel shows the density depletion on H2 and on π-orbitals of the carbon atom in the para and meta positions. The density change can be characterized as an electron transfer from the hydrogen molecule to an empty orbital on the metal and can be classified as a Kubas-like interaction. However, this interaction differs from a typical Kubas interaction and that observed in “Be_2_-benzene”. The NOCV charge flow channels for “Mg_2_-benzene” shows that only one atom from H_2_ is involved in the Kubas-type interaction. This effect is probably caused by hydrogen polarization. The electrons from the sigma-orbital are moved toward H1 and, as a consequence, the Kubas-like interaction involves only one hydrogen from the H_2_ molecule. This interaction, which involves the p-orbital from the metal and one hydrogen atom will be referred to here as a “polarized Kubas-like” interaction. The second NOCV charge flow channel reveals density depletion on H1, and a density increase on H2 and π orbitals (mainly on carbon in the para position). The channel describes the interactions of hydrogen with π orbitals. The third channel shows an increase in density on the hydrogen molecule with very diffuse density depletion behind H_2_ (not shown on the picture). This phenomenon can be characterized as a back-donation.

**Table 5:** Interaction energy decomposition of hydrogen energy adsorption and ETS-NOCV analysis

	C_5_H_6_Be_2_			C_5_H_6_Mg_2_			C5H6Ca2			
	1^st^ H_2_	2^nd^ H_2_/Be_2_	2^nd^ H_2_/Be	1^st^ H_2_	2^nd^ H_2_/Mg_2_	2^nd^ H_2_/Mg	1^st^ H_2_ @Ca_up_	2^nd^ H_2_ @Ca_up_	1^st^ H_2_ @Ca_down_	2^nd^ H_2_ @Ca_down_
ΔE_MP2_	−5.10	−1.94	−1.32	−3.81	−2.26	−0.60	−3.76	−1.42	−1.65	−1.21
E_el_	−13.60	−11.42	−2.33	−7.62	−4.94	−3.41	−7.78	−3.25	−2.16	−2.65
E_ex_	29.59	25.54	4.48	14.70	9.72	6.03	13.36	4.73	4.12	3.65
E_del_	−18.32	−14.70	−1.29	−7.17	−3.94	−2.98	−6.95	−2.20	−1.29	−1.89
ΔE_HF_	−2.34	−0.58	0.86	−0.09	0.84	−0.36	−1.37	−0.72	0.68	−0.89
E_MP2_	−2.76	−1.36	−2.18	−3.72	−3.10	−0.24	−2.39	−0.71	−2.32	−0.32
E_orb_	−22.40	−18.94	−2.19	−8.55	−4.87	−3.82	−7.58	−3.09	−1.69	−2.62
M ← H_2_^c^	−13.51^b^	−10.67^a^	–	–	–	−2.29	−2.19^b^	−1.78^a^	–	−1.47^a^
MH ← H_2_^d^	–	–	–	–	–	–	–	−0.93	–	−0.87
M ← H_2_ → C^e^	–	–	–	−5.11	−2.77	–	−4.94	–	–	–
C ← H_2_^f^	–	–	−1.27	−1.69	−0.95	–	–	–	−1.08	–
Back-donation^g^	−5.45 −2.16	−5.45 −1.75	–	−1.24	−0.81	−1.19	–	–	–	–
*E*_Pauli_	30.37	28.22	4.05	13.70	8.93	6.78	10.48	4.53	3.62	3.91
*E*_elst_	−13.87	12.44	−2.39	−7.74	−5.07	−3.35	−6.81	−3.06	−2.44	−2.56

^a^Asymmetric interaction

^b^Symmetric interaction

^c^Electron transfer from hydrogen molecule to metal atom

^d^Transfer to MH bond

^e^M ← H_2_ → C – electron transfer to metal and carbon atoms

^f^C ← H_2_ – electron transfer to carbon atom(s)

^g^Back-donation – electron transfer to H_2_ molecule

Adsorption of the second hydrogen on the Mg_2_ center takes place on the second magnesium atom on the opposite site of the molecule but in a similar manner to the first. The adsorption energy is significantly lower compared to the adsorption of the first H_2_ (2.3 kcal mol^−1^). The energy decomposition shows that absolute values of all energy components are lower and their proportions are changed slightly. The dispersion attractions are more important in binding of the second hydrogen, whereas the delocalization term is less significant. ETS-NOCV analysis showed the same charge flow channels but its contribution to the complex stabilization is lower. The adsorption energy of the second hydrogen on the same magnesium atom of “Mg_2_-benzene” is as low as 0.6 kcal mol^−1^. The interaction energy decomposition reveals that the adsorption is controlled by electrostatic and delocalization terms. ETS-NOCV charge flow channels show that the delocalization term is dominated by the Kubas interaction and the corresponding back-donation.

In summary, the replacement of Be_2_ by Mg_2_ changes structure, orbitals, and aromaticity of compounds only slightly. However, the hydrogen adsorption mechanism on C_5_H_5_Mg_2_ differs significantly from that on C_5_H_5_Be_2_.

### “Ca_2_-benzene”

The structure of “Ca_2_-benzene” (Table 2) differs significantly from that of “Be_2_-benzene” and “Mg_2_-benzene”. The molecule no longer possesses a C_2_ axis of symmetry and the calcium atoms are not symmetrically equivalent. The first calcium atom (denoted as Ca_down_) is close to the plane and interacts with two neighboring carbon atoms, whereas the second calcium atom (Ca_up_) lies above the C_5_H_5_ plane. The Ca_up_ atom also interacts with π orbitals of other carbon atoms. The AIM analysis (Table S5) shows close shell bonding interactions between Ca_up_ and carbon in the para position (C_para_). Due to this interaction, C_para_ lies slightly above the C_5_H_5_ plane. The orbital contours indicates that the bonding interactions between metal atoms and neighboring carbons and hydrogen are similar to these in “Mg_2_-benzene”. However, the character of the third molecular orbital differs from that of “Mg_2_-benzene” and confirms that the π orbital(s) from other carbon atoms are involved in calcium binding. The calcium interactions with π orbitals destroy the symmetry and shape of the π orbitals. The first orbital spans all the carbon atoms; however, it has no planar symmetry. The second orbital covers C–C and C–H bonds and differs drastically from those in benzene. The third orbital possesses a localized character typical of isolated p orbitals. In summary, π orbitals possess partially delocalized character but the interaction with Ca_up_ atom destroys their aromaticity.

Values of all NICS indices are close to zero indicating that the compound is not aromatic. In comparison to “Mg_2_-benzene”, “Ca_2_-benzene” has lower electron density and lower laplacian of electron density in RCP, which confirms its lower aromatic character. However, the aromatic stabilization index leads to the opposite conclusion. The ASE value for “Ca_2_-benzene” is over two times higher than that for “Mg_2_-benzene”. This leads to the interpretation that “Ca_2_-benzene” is more aromatic that “Mg_2_-benzene”. The ASE index represents the difference between an aromatic compound and its non-aromatic isomer; normally, this energy corresponds to the aromatic stabilization. In the case of “Ca_2_-benzene”, the calcium atom interacts with π orbitals, thus the ASE energy can be considered as the sum of aromatic stabilization and Ca–π interactions. Therefore, the picture drawn by NICS indices seems to be the most reliable. Hence, one can conclude that Ca_2_-benzene has negligible aromatic character.

Since calcium atoms in “Ca_2_-benzene” are not symmetrically equivalent, their interactions with hydrogen differ. The upper calcium adsorbs the first hydrogen with an energy of 3.8 kcal mol^−1^, which is equal to adsorption of the first H_2_ on “Mg_2_-benzene”. The energy decomposition shows that the nature of adsorption is similar in both complexes; however, the dispersion component is less significant in the Ca_2_-benzene case. Two ETS-NOCV charge flow channels are responsible for 94 % of delocalization energy. The first channel shows a density increase on the hydrogen atom closer to Ca (H1) and between H1 and calcium. Electron depletion is observed on the hydrogen close to the aromatic ring (H2) and on the π orbitals of the carbon atom in the para position (C4) and in ortho positions. This channel also reveals the density increase between H2 and C4 atoms. The interactions constituting the first channel can be summarized as polarized Kubas-like, supported by H_2_–π interactions. The adsorption energy of the second H_2_ on Ca_up_ is 1.4 kcal mol^−1^ and electrostatic interaction constitutes the main attractive term. The charge flow channel reveals that the delocalization term (which is responsible for 36 % of attractive interactions) is controlled by the polarized Kubas interaction.

The calcium, which is located close to the plane of the ring, can adsorb up to two hydrogen molecules but the energy is as low as 1.6 and 1.2 kcal mol^−1^. The first H_2_ is adsorbed above the aromatic ring and the interaction is controlled by electrostatic and dispersion contributions. The delocalization is responsible for only 22 % of attractive interactions. ETC-NOCV analysis indicated the lack of electronic interactions between hydrogen and the calcium atom. Such interactions were observed between H_2_ and π orbitals. The adsorption of the second H_2_ was controlled by electrostatic attractions, which were responsible for 49 % of attractive interactions, whereas delocalization and electron correlation contributions constituted 35 % and 16 %, respectively. The delocalization term is dominated by polarized Kubas, which is responsible for 89 % of the delocalization energy.

### “BeMg-benzene”

The structure of “BeMg-benzene” is similar to that of “Be_2_-benzene” and “Mg_2_-benzene”, i.e., all carbon and hydrogen atoms lie on a plane and the metal atoms are on the perpendicular axis. Distances of metal atoms from the plane are not equal: beryllium is located 0.5 Å below whereas magnesium lies 1.6 Å above it. In “BeMg-benzene”, the Mg–C and Mg–H bonds are shorter compared to Mg_2_-benzene, which may indicate that the Mg_2_ center is slightly too large to fit in the carbon atom position. However, replacement of Mg with the smaller Be atom does not affect the MOs. Orbitals involved in metal binding as well as π orbitals have the same character in “BeMg-benzene” as in pure compounds.

The NICS value in the RCP is close to zero, whereas 1 Å above RCP is negative, which is similar to the case for C_5_H_6_Be_2_ and C_5_H_6_Mg_2_. Other AIs suggest that “BeMg-benzene” is more aromatic that “Mg_2_-benzene”. Absolute values of electron density, density laplacian, and electron energy in RCP are approximately one-third higher that those of “Mg_2_-benzene”. Similarly, the SA index of “BeMg-benzene” is one-fourth lower compared to “Mg_2_-benzene” whereas ASE is over three times higher. Based on the results presented, one can conclude that the replacement of magnesium with beryllium in “Mg_2_-benzene” stabilized the structure and increased its aromatic character.

“BeMg-benzene” can adsorb the first hydrogen with relatively high energy of 6.5 kcal mol^−1^. H_2_ is located over the carbon ring close to magnesium. Adsorption is controlled by delocalization and electrostatic terms that are responsible for 44 % and 40 % of attractive interactions, respectively. The electron correlation constitutes only 16 %. The ETS-NOCV analysis revealed that three charge flow channels significantly stabilize the complex. The first, which can be characterized as polarized Kubas, is supported by interaction with π orbitals. This interaction is responsible for 60 % of delocalization energy. The second channel (22 % of delocalization energy) can be classified as a hydrogen interaction with π orbitals, whereas back-donation—the third channel—constitutes 13 % of overall delocalization energy. The adsorption energy of the second hydrogen on Mg amounts to 0.7 kcal mol^−1^ only. This interaction is also controlled by electrostatic and delocalization terms but details of the delocalization attraction are different. The main delocalization component (62 % of overall energy) is represented by Kubas interactions involving both hydrogen atoms (although not in equal proportions). The back-donation, which causes an increase of density on the adsorbed hydrogen, is responsible for 24 % of delocalization energy.

The hydrogen adsorption on beryllium in “BeMg-benzene” is low; the first H_2_ can be bound with an energy of 1.2 kcal mol^−1^, whereas the second is bound with 0.2 kcal mol^−1^ only. Adsorption is controlled by electron correlation (46 % of attractive contribution) and electrostatics (37 %). Analysis of the delocalization term shows no interaction with the metal center, which rather comes from interactions with π orbitals. Similarly, the second hydrogen is bound by delocalization and electrostatic terms. It is worth noting that the hydrogen adsorption properties of beryllium in “BeMg-benzene” decreases dramatically compared to those of “Be_2_-benzene”. A possible explanation for this fact is the high electron density (which can be seen on the bonding orbital) and low atomic charge on the Be atom. The NBO charge on Be in “BeMg-benzene” is only 0.540, whereas in “Be_2_-benzene” it is 0.822 electron.

### “BeCa-benzene” and “MgCa-benzene”

In “BeCa-benzene” and “MgCa-benzene” compounds, the smaller metal atom is close to the C_5_H_5_ plane. Being above the ring, the calcium atom interacts not only with neighboring carbons but also with π electrons of other carbon atoms. The carbon–calcium bond length is 2.527 Å in “BeCa-benzene” and 2.507 Å in “MgCa-benzene”. The interatomic distances between calcium and carbon in the meta position for “BeCa-benzene” and “MgCa-benzene” are 2.705 Å and 2.725 Å, whereas the distances to carbon in the para position are 2.682 Å and 2.710 Å, respectively. AIM analysis in both cases indicates Ca–C_para_ bonding interactions. Bonding orbitals in “BeCa-benzene” have no π character whereas the third orbital involved in bonding metals has a partial π character. The contour of π orbitals indicates that, in both compounds, the first orbital is delocalized over all carbons. The second π orbital in both compounds has one nodal plane, whereas the symmetry and shape of both compounds is different. “BeCa-benzene” possesses a typical π orbital that spans symmetricaly over carbon atoms in ortho and meta positions, whereas in “MgCa-benzene” this orbital also covers the C–H bond. The third orbital in both compounds reveals interactions with metal. NICS indices show a lack of aromaticity in both compounds.

Like “Ca_2_-benzene” and “BeMg-benzene”, the adsorption of hydrogen on “BeCa-benzene” and “MgCa-benzene” proceeds rather on the heavier metal located above the carbon ring. The calcium atom in “BeCa-benzene” can bind two hydrogen atoms with energies of 4.5 and 2.5 kcal mol^−1^, respectively. Both interactions are controlled by electrostatic and delocalization terms. Orbital interactions reveal that 64 % of the delocalization contribution comes from polarized Kubas interactions supported by interactions with the π orbital of carbon in the para position, and 31 % from the “classical” Kubas orbital overlapping. The second hydrogen is located above the Ca–H bond. ETS-NOCV charge flow channels reveal that 93 % of the delocalization interaction came from polarized Kubas interactions. The mechanism of hydrogen adsorption on the Ca atom in “MgCa-benzene” is identical but the overall adsorption energy is lower by 3.9 and 1.9 kcal mol^−1^, respectively.

The lighter element (Be and Mg) in the compounds presented can adsorb up to two H_2_ molecules but with interaction energies as low as 1.1–1.4 kcal mol^−1^. In “BeCa-benzene”, the hydrogen adsorption energy is virtually the same for the first and second H_2_ molecule; however, the energy components reveals a dramatically different mechanism. The energy decomposition (Table 6) indicates significant delocalization and electrostatic interactions (both above 10 kcal mol^−1^) between the first H_2_ and “BeCa-benzene”. ETS-NOCV charge flow channels (Tables 7–12) show that the delocalization interaction comes from Kubas interactions involving both hydrogen atoms (58 % of delocalization energy) and two polarized Kubas interaction between one hydrogen atom from the H_2_ molecule and beryllium (22 % and 16 % of the delocalization term). Adsorption of the second hydrogen has a similar overall energetic effect (1.3 kcal mol^−1^) but the mechanism is different. The main attractive term comes from electron correlation, which is responsible for 48 % of the attractive energy, while the delocalization term constitutes only 17 % of overall attractions. ETS-NOCV decomposition shows no significant interactions between hydrogen and beryllium, and indicates that the delocalization term comes from interactions with π orbitals. A similar situation is observed in “MgCa-benzene”, where adsorption of the first hydrogen is controlled by electrostatic and delocalization terms. The delocalization component comes from Kubas interactions supported by interactions with π orbitals (the first charge flow channel) and polarized Kubas interaction. Adsorption of the second H_2_ is controlled by electron correlation and electrostatic contributions.

**Table 6:** Interaction energy decomposition of hydrogen adsorption energy and the ETS-NOCV analysis.Definitions as in Table 5

	C_5_H_6_BeMg				C_5_H_6_BeCa				C_5_H_6_MgCa			
	1^st^H_2_ @Be	2^nd^ H_2_ @Be	1^st^ H_2_ @Mg	2^nd^ H_2_ @Mg	1^st^ H_2_ @Be	2^nd^ H_2_ @Be	1^st^ H_2_ @Ca	2^nd^ H_2_ @Ca	1^st^ H_2_ @Mg	2^nd^ H_2_ @Mg	1^st^ H_2_ @Ca	2^nd^ H_2_ @Ca
ΔE_MP2_	−1.17	−0.25	−6.51	−0.73	−1.13	−1.31	−4.51	−2.53	−1.37	−1.34	−3.94	−1.87
E_el_	−1.79	−0.53	−11.36	−3.74	−10.65	−1.46	−8.51	−3.63	−4.24	−1.63	−7.56	−3.38
E_ex_	3.66	1.11	21.83	6.50	22.73	2.84	14.58	4.89	6.73	3.27	12.78	4.80
E_del_	−0.82	−0.21	−12.55	−3.48	−10.52	−0.70	−8.10	−3.00	−3.61	−0.84	−6.83	−2.57
ΔE_HF_	1.05	0.38	−2.08	−0.72	1.57	0.69	−2.03	−1.74	−1.12	0.80	−1.61	−1.15
E_MP2_	−2.22	−0.62	−4.42	−0.10	−2.69	−2.00	−2.48	−0.79	−0.24	−2.14	−2.33	−0.72
E_orb_	−1.68	−0.80	−14.45	−4.42	−13.85	−1.22	−8.89	−4.00	−4.84	−1.35	−7.49	−3.42
M ← H_2_	–	–	–	−2.74^a^	−7.99^b^	–	−2.74^b^	−2.62^a^	−2.84^b^	–	−2.34^b^	−2.21^a^
					−3.00^a^							
MH ← H_2_	–	–	–	−0.40	−2.12	–	–	−1.09	–	–	–	−0.86
M ← H_2_ → C	–	–	−8.74	–	–	–	−5.75	–	–	–	−4.76	–
			−3.16									
E_pauli_	3.26	1.10	20.62	7.25	22.71	2.49	11.08	4.40	7.76	2.91	9.87	4.49
E_elst_	−1.94	−0.50	−11.55	−3.73	−10.67	−1.72	−7.39	−3.50	−4.21	−1.93	−6.60	−3.21

^a^Asymmetric interaction

^b^Symmetric interaction molecule to metal atom

**Table 7:**
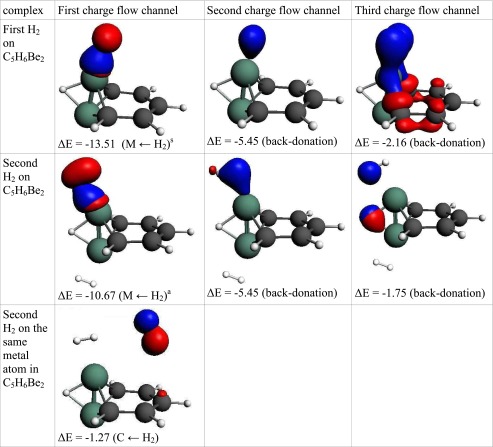
The interaction of hydrogen(s) with “Be_2_-benzene”. NOCV charge flow channels (*blue/red contours* represent accumulation/depletion electron density), corresponding energy (in kcal mol^−1^), and flow type. The flow channels are ordered by decreasing energy

a – asymmetric interaction, s – symmetric interaction, M ← H_2_ – electron transfer from hydrogen molecule to metal atom, MH ← H_2_ – electron transfer to MH bond, M ← H_2_ → C – electron transfer to metal and carbon atoms, C ← H_2_ – electron transfer to carbon atom(s), Back-donation – electron transfer to H_2_ molecule

**Table 8:**
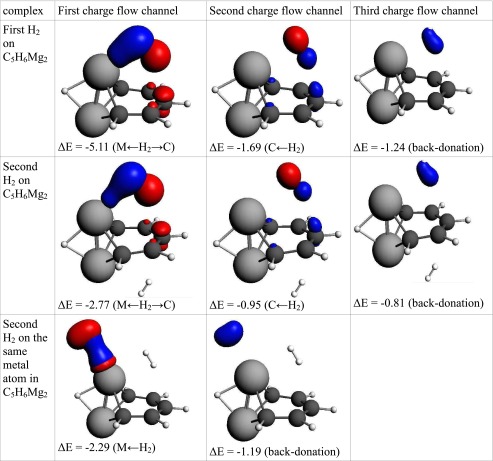
The interaction of hydrogen(s) with “Mg_2_-benzene”. NOCV charge flow channels (*blue/red contours* represent accumulation/depletion electron density), corresponding energy (in kcal mol^−1^), and flow type. The flow channels are ordered by decreasing energy

a – asymmetric interaction, s – symmetric interaction, M ← H_2_ – electron transfer from hydrogen molecule to metal atom, MH ← H_2_ – electron transfer to MH bond, M ← H_2_ → C – electron transfer to metal and carbon atoms, C ← H_2_ – electron transfer to carbon atom(s), Back-donation – electron transfer to H_2_ molecule

**Table 9:**
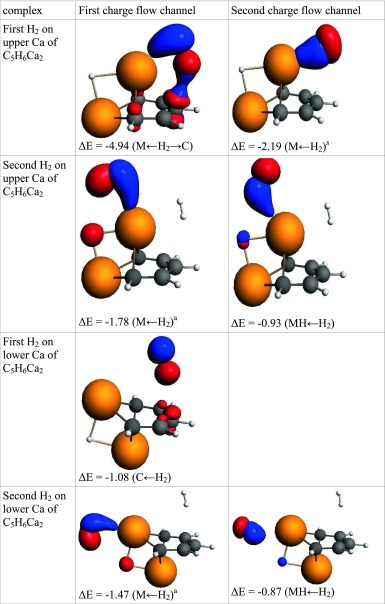
The interaction of hydrogen(s) with “Ca_2_-benzene”. NOCV charge flow channels (*blue/red contours* represent accumulation/depletion electron density), corresponding energy (in kcal mol^−1^), and flow type. The flow channels are ordered by decreasing energy

a – asymmetric interaction, s – symmetric interaction, M ← H_2_ – electron transfer from hydrogen molecule to metal atom, MH ← H_2_ – electron transfer to MH bond, M ← H_2_ → C – electron transfer to metal and carbon atoms, C ← H_2_ – electron transfer to carbon atom(s), Back-donation – electron transfer to H_2_ molecule

**Table 10:**
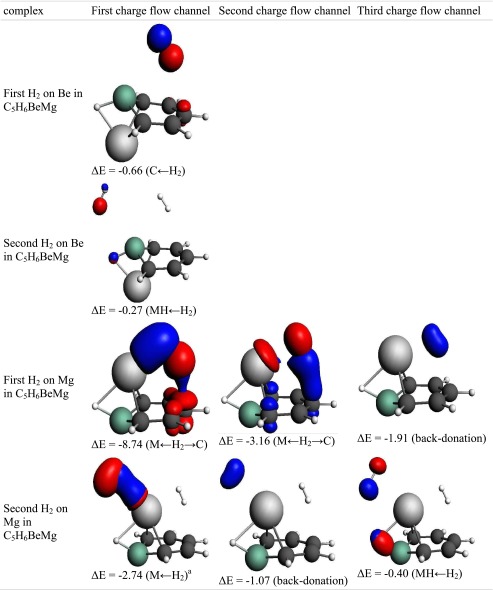
The interaction of hydrogen(s) with “BeMg-benzene”. NOCV charge flow channel (*blue/red contours* represent accumulation/depletion electron density), corresponding energy (in kcal mol^−1^), and flow type. The flow channels are ordered by decreasing energy

a – asymmetric interaction, s – symmetric interaction, M ← H_2_ – electron transfer from hydrogen molecule to metal atom, MH ← H_2_ – electron transfer to MH bond, M ← H_2_ → C – electron transfer to metal and carbon atoms, C ← H_2_ – electron transfer to carbon atom(s), Back-donation – electron transfer to H_2_ molecule

**Table 11:**
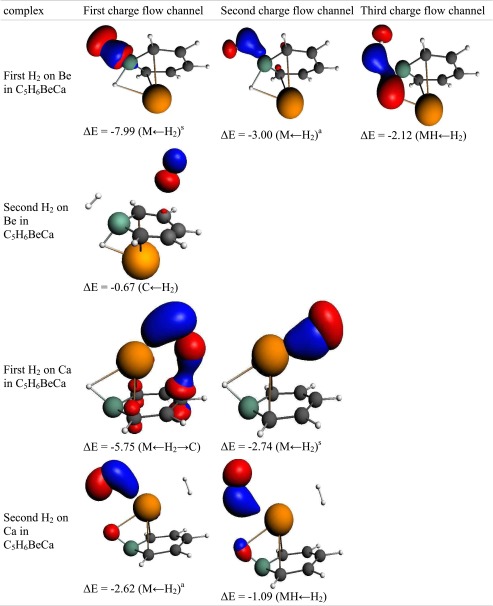
The interaction of hydrogen(s) with “BeCa-benzene”. NOCV charge flow channel (*blue/red contours* represent accumulation/depletion electron density), corresponding energy (in kcal mol^−1^) and flow type. The flow channels are ordered by decreasing energy

a – asymmetric interaction, s – symmetric interaction, M ← H_2_ – electron transfer from hydrogen molecule to metal atom, MH ← H_2_ – electron transfer to MH bond, M ← H_2_ → C – electron transfer to metal and carbon atoms, C ← H_2_ – electron transfer to carbon atom(s), Back-donation – electron transfer to H_2_ molecule

**Table 12:**
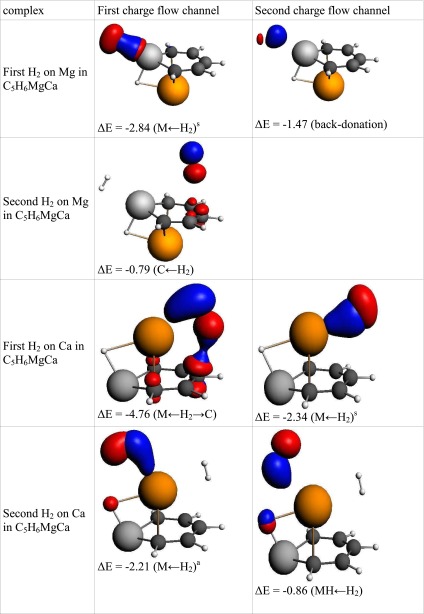
The interaction of hydrogen(s) with “MgCa-benzene”. NOCV charge flow channel (*blue/red contours* represent accumulation/depletion electron density), corresponding energy (in kcal mol^−1^) and flow type. The flow channels are ordered by decreasing energy

a – asymmetric interaction, s – symmetric interaction, M ← H_2_ – electron transfer from hydrogen molecule to metal atom, MH ← H_2_ – electron transfer to MH bond, M ← H_2_ → C – electron transfer to metal and carbon atoms, C ← H_2_ – electron transfer to carbon atom(s), Back-donation – electron transfer to H_2_ molecule

## Conclusions

Beryllium and magnesium can form metallabenzene-type compounds of general formula C_5_H_6_M_2_ exhibiting some aromatic character. The aromatic character comes from the C_5_H_5_^−^ fragment rather than from the whole molecule. The heavier group 2 metals (e.g., calcium) are too large to fit in the carbon position, hence C_5_H_6_Ca_2_ has insignificant aromaticity. Aromatic properties of benzene analogues with two different metals depend on the atom size and relative difference in the size, hence the highest aromaticity is observed in C_5_H_6_BeMg, whereas compounds with calcium atom do not exhibit aromatic character.

Aromatic indices for such compounds should be interpreted with caution. The values obtained result not only from aromatic properties but also from the metal type. AIM-based AIs give preference to more electronegative atoms. The ASE index for the compounds presented measure the sum of aromatic stabilization and metal–π interaction rather than the aromaticity alone.

C_5_H_6_M_2_-type compounds can adsorb a single hydrogen molecule with energy up to 6.5 kcal mol^−1^, depending on the metal type. Such a value is sufficient for commercial applications. Energy decomposition shows that adsorption is controlled by delocalization and electrostatic contributions. The adsorption mechanism in Be_2_-benzene involves only metal atoms; however, in other compounds the carbon atom also takes part in the hydrogen binding. NOCV charge flow channels reveal that one hydrogen atom from the H_2_ molecule interacts with π orbitals of the C_5_H_5_^−^ ligand whereas the second hydrogen atom interacts with an empty orbital of the metal.

The aromatic character of compounds with two different metals (C5H6MM’) lies between properties of systems with the same metal type (C_5_H_5_M_2_ and C_5_H_5_M’_2_). However, the interaction with hydrogen for such compounds is stronger than that for C_5_H_5_M_2_ or C_5_H_5_M’_2_ and adsorption occurs on heavier metals. This tendency is visible, especially in C_5_H_6_BeMg. In “BeCa-benzene” and “MgCa-benzene”, this effect is rather low and the adsorption energy is comparable to that of “Ca_2_-benzene”.

## Electronic supplementary material

Below is the link to the electronic supplementary material.

ESM 1 (DOCX 1905 kb)

## References

[1] Katritzky AR, Ramsden CA, Joule JA, Zhdankin VV (2010). Handbook of heterocyclic chemistry.

[2] Masui H (2001). Coord Chem Rev.

[3] Bleeke JR (2001). Chem Rev.

[4] Feixas F, Matito E, Poater J, Solà M (2013). WIREs Comput Mol Sci.

[5] Li X, Kuznetsov AE, Zhang HF, Boldyrev AI, Wang LS (2003). Science.

[6] Badri Z, Pathak S, Fliegl H, Rashidi-Ranjbar P, Bast R, Marek R, Foroutan-Nejad C, Ruud K (2013). J Chem Theory Comput.

[7] Kruszewski J, Krygowski TM (1972). Tetrahedron Lett.

[8] Schleyer PR, Maerker C, Dransfeld A, Jiao H, Hommes NJRE (1996). J Am Chem Soc.

[9] Bader RFW (1990). Atoms in molecules.

[10] George P, Trachtman M, Bock CW, Brett AM (1976). J Chem Soc Perkin Trans.

[11] Schleyer PR, Pühlhofer F (2002). Org Lett.

[12] Palusiak M, Krygowski TM (2007). Chem Eur J.

[13] Ebrahimi AA, Ghiasi R, Foroutan-Nejad C (2010). J Mol Struct THEOCHEM.

[14] Matito E, Poater J, Sola M, Matta CF, Boyd RJ (2007). Aromaticity analysis by means of the quantum theory of atoms in molecules. The quantum theory of atoms in molecules.

[15] Poater J, Fradera X, Duran M, Sola M (2003). Chem Eur J.

[16] Matito E, Duran M, Sola M (2005). J Chem Phys.

[17] Noorizadeh S, Shakerzadeh E (2010). Phys Chem Chem Phys.

[18] Shannon CE (1948). Bell Syst Tech J.

[19] Jia G (2013). Organometallics.

[20] Chen J, Jia G (2013). Coord Chem Rev.

[21] Fernandez I, Frenking G (2007). Chem Eur J.

[22] Roszak R, Roszak S, Majumdar D, Kuchta B, Firlej L, Leszczynski J (2014). J Phys Chem A.

[23] Møller C, Plesset M (1934). Phys Rev.

[24] Prascher BP, Woon DE, Peterson KA, Dunning TH (2011). Wilson AK. Theor. Chem. Accounts.

[25] Cheeseman JR, Trucks GW, Keith TA, Frisch MJ (1996). J Chem Phys.

[26] Frisch MJ, Trucks GW, Schlegel HB, Scuseria GE, Robb MA, Cheeseman JR, Scalmani G, Barone V, Mennucci B, Petersson GA et al. (2013) Gaussian 09, Revision D. 01. Gaussian, Inc., Wallingford, CT

[27] Gora RW, Bartkowiak W, Roszak S, Leszczynski J (2004). J Chem Phys.

[28] Schmidt MW, Baldridge KK, Boatz JA, Elbert ST, Gordon MS, Jensen JH, Koseki S, Matsunaga N, Nguyen KA, Su S, Windus TL, Dupuis M, Montgomery JA (1993). J Comput Chem.

[29] Gora, RW (2009) EDS package v. 2.8.3, Wroclaw

[30] Mitoraj M, Michalak A, Ziegler T (2009). J Chem Theory Comput.

[31] Ziegler T, Rauk A (1977). Theor Chim Acta.

[32] Michalak A, Mitoraj M, Ziegler T (2008). J Phys Chem A.

[33] van Lenthe E, Baerends EJ (2003). J Comput Chem.

[34] te Velde G, Bickelhaupt FM, van Gisbergen SJA, Fonseca Guerra C, Baerends EJ, Snijders JG, Ziegler T (2001). J Comput Chem.

[35] Fonseca Guerra C, Snijders JG, te Velde G, Baerends EJ (1998). Theor Chem Acc.

[36] ADF (2013) SCM, Theoretical Chemistry, Vrije Universiteit, Amsterdam, The Netherlands, http://www.scm.com

[37] Jmol: an open-source Java viewer for chemical structures in 3D. http://www.jmol.org/

[38] ADF-GUI 2013, SCM, Amsterdam, The Netherlands, http://www.scm.com

[39] Lu T, Chen F (2012). J Comp Chem.

[40] Kubas GJ (2001). Metal dihydrogen and σ-bond complexes: structure, theory, and reactivity.

