# Supplementary material for: s-Block metallabenzene: aromaticity and hydrogen adsorption
Source: J Mol Model. 2015 Jan 29;21(2):28. doi: 10.1007/s00894-014-2552-6 (PMC4309903; doi:10.1007/s00894-014-2552-6)
Supplement: Supplementary file 1 — (DOCX 1905 kb) [file 894_2014_2552_MOESM1_ESM.docx]

**Supporting information**

**s-block metallabenzene: aromaticity and hydrogen adsorption**

Table S1: The aromaticity of C_5_H_6_X and C_5_H_5_^-^

| Compound | C_5_H_6_Ge | C_5_H_6_Si | benzene | C_5_H_5_^-^ | C_5_H_5_^-a^ | C_5_H_5_^-b^ |
| --- | --- | --- | --- | --- | --- | --- |
| NICS(0) | -7.82 | -7.96 | -7.53 | -12.26 | -18.98 | -6.07 |
| NICS(0)_zz_ | -8.56 | -11.79 | -13.25 | -17.37 | -24.76 | 20.29 |
| NICS(1) | -9.02 | -8.98 | -9.95 | -9.59 | -5.90 | -5.28 |
| NICS(1)_ZZ_ | -24.32 | -24.90 | -29.50 | -34.95 | -22.99 | 8.849 |
| density | 0.0150 | 0.0158 | 0.0210 | 0.0474 | 0.0156 | 0.0107 |
| laplacian | 0.0858 | 0.0911 | 0.1568 | 0.3193 | 0.0841 | 0.0490 |
| H | 0.0041 | 0.0043 | 0.0080 | 0.0054 | 0.0046 | 0.0025 |
| G | 0.0173 | 0.0184 | 0.0299 | 0.0744 | 0.0163 | 0.0098 |
| V | -0.0132 | -0.0141 | -0.0211 | -0.0691 | -0.0046 | -0.0074 |
| SA *10^2^ | 4.41 | 6.31 | 0.00 | 0.00 | 15.27 | 17.90 |
| ASE [ kcal/mol] | 32.8 | 33.2 | 37.0 | 31.5 | --- | --- |

a – in C_5_Be_2_H_6_ geometry, b – in C_5_Mg_2_H_6_ geometry

Table S2: Electron density properties in C_orto_-C_orto_ BCP

| Compound | C_5_H_5_^-a^ | C_5_H_5_^-b^ |
| --- | --- | --- |
| Electron density | 0.0233 | 0.0124 |
| laplacian | 0.0356 | 0.0360 |
| H | 0.0018 | 0.0013 |
| G | 0.0070 | 0.0077 |
| V | -0.0053 | -0.0064 |

a – in C_5_H_6_Be_2_ geometry, b – in C_5_H_6_Mg_2_ geometry

Table S3: Aromatic orbitals of silabenzene and germabenzene. The orbitals are ordered by increasing energy (the first has the lowest energy)

| Compound | First π orbital | Second π orbital | Third π orbital |
| --- | --- | --- | --- |
| Silabenzene | 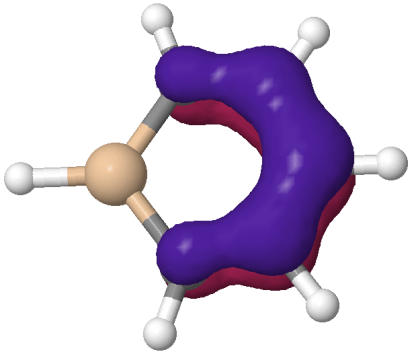 | 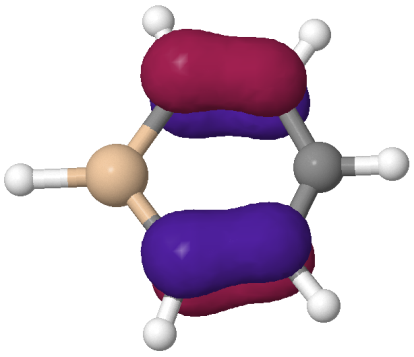 | 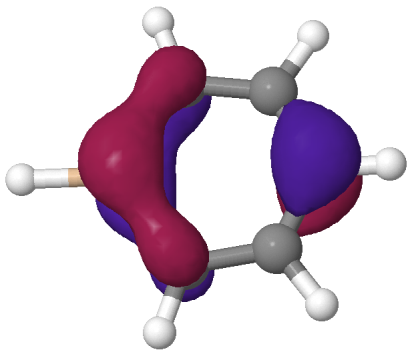 |
| Germabenzene | 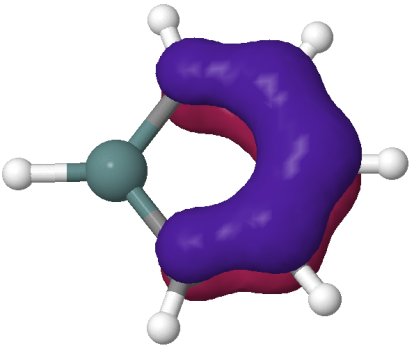 | 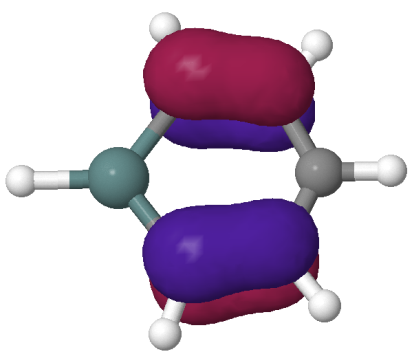 | 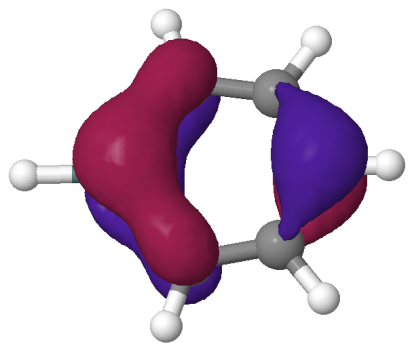 |

Table S4: Hydrogen adsorption energy decomposition

|  | C_6_H_6_ | C_5_H_6_Si | C_5_H_6_Ge |
| --- | --- | --- | --- |
| ΔE_MP2_ | -0.88 | -0.95 | -0.95 |
| E_el_ | -1.17 | -1.22 | -1.23 |
| E_ex_ | 2.64 | 2.73 | 2.75 |
| E_del_ | -0.43 | -0.52 | -0.51 |
| ΔE_HF_ | 1.04 | 1.00 | 1.01 |
| E_MP2_ | -1.92 | -1.95 | -1.96 |
| E_orb_ | -1.03 | -0.99 | -1.14 |
| E_pauli_ | 2.44 | 2.51 | 2.49 |
| E_elst_ | -1.43 | -1.45 | -1.45 |

Table S5: Electron density properties in BCPs

| bond | property | C_5_H_6_Be_2_ | C_5_H_6_Mg_2_ | C_5_H_6_Ca_2_ | C_5_H_6_BeMg | C_5_H_6_BeCa | C_5_H_6_MgCa |
| --- | --- | --- | --- | --- | --- | --- | --- |
| M-M |  | No BCP | No BCP | No BCP | No BCP | No BCP | No BCP |
| M-H_M_ | Laplacian | 0.2758 | 0.0853 | 0.0853 | 0.2579  0.1094 | 0.2450  0.0919 | 0.1528  0.0809 |
|  | Density | 0.0595 | 0.0266 | 0.0277 | 0.0661  0.0284 | 0.0596  0.0251 | 0.0337  0.0247 |
|  | H | -0.0118 | -0.0016 | 0.0005 | -0.0166  -0.0005 | -0.0126  0.0005 | 0.0013  0.0011 |
|  | Ellipticity | 3.2e-05 | -1.0975 | 0.0452 | 0.1146  0.9234 | 0.1837  3.0069 | 0.0233  0.1300 |
| M-C_orto_ | Laplacian | 0.3325 | 0.1900 | 0.1331  0.1328 | 0.3630  0.1494 | 0.3892  0.1265 | 0.2280  0.1280 |
|  | Density | 0.0712 | 0.0385 | 0.0369  0.0408 | 0.0776  0.0342 | 0.0810  0.0360 | 0.0426  0.0360 |
|  | H | -0.0143 | 0.0027 | 0.0011  4.42e-05 | -0.0157  0.0013 | -0.0160  0.0010 | 0.0038  0.0012 |
|  | Ellipticity | 0.1262 | 0.1591 | 0.2126  0.1196 | 0.0306  0.5277 | 0.0567  0.7024 | 0.1072  0.2726 |
| C_orto_-H_M_ | Laplacian | 0.0360 | 0.0254 | No BCP | No BCP | No BCP | No BCP |
|  | Density | 0.0537 | 0.0209 |  |  |  |  |
|  | H | -0.0220 | -0.0040 |  |  |  |  |
|  | Ellipticity | 0.2974 | 1.9507 |  |  |  |  |
| C_orto_-C_orto_ | Laplacian | 0.0457 | 0.0355 | No BCP | No BCP | No BCP | No BCP |
|  | Density | 0.0577 | 0.0246 |  |  |  |  |
|  | H | -0.0212 | -0.0041 |  |  |  |  |
|  | Ellipticity | 1.6202 | 1.3691 |  |  |  |  |
| M-C_para_ | Laplacian | No BCP | No BCP | 0.0880 | No BCP | 0.0902 | 0.0870 |
|  | Density |  |  | 0.0207 |  | 0.0230 | 0.0207 |
|  | H |  |  | 0.0032 |  | 0.0028 | 0.0032 |
|  | Ellipticity |  |  | 1.2349 |  | 0.9785 | 1.1887 |

Table S6: Structures of C_5_H_6_M_2_ in complexes with hydrogen(s)

| compounds | Complex with 1 H_2_ | Complex with 2 H_2_ | Complex with 2 H_2_ on the same metal atom |
| --- | --- | --- | --- |
| C_5_H_6_Be_2_ | 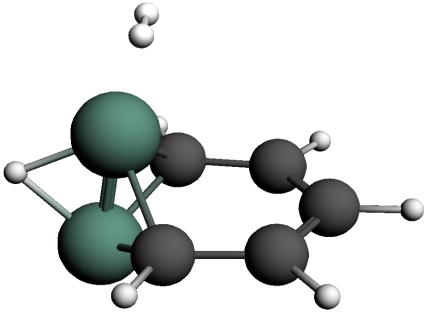 | 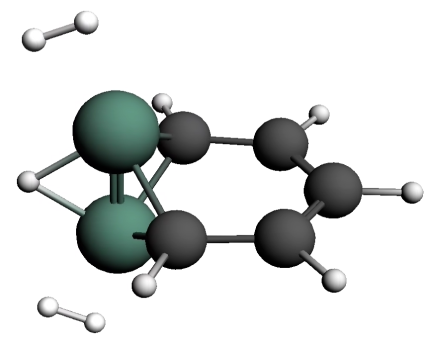 | 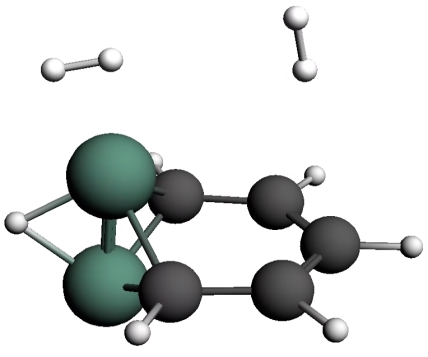 |
| C_5_H_6_Mg_2_ | 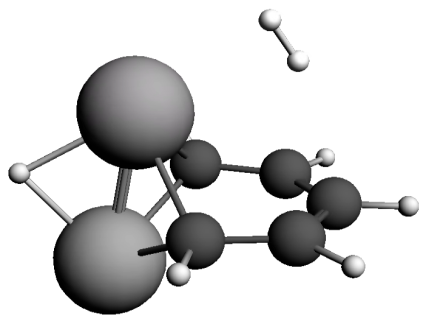 | 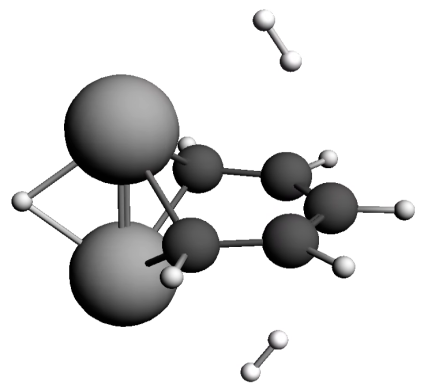 | 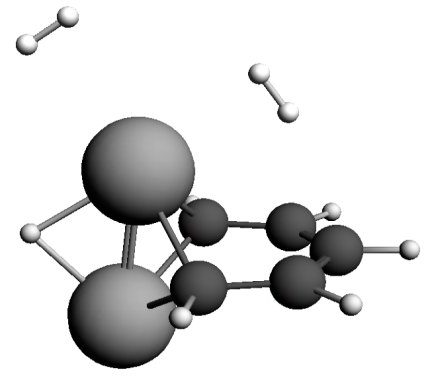 |
| C_5_H_6_Ca_2_ | 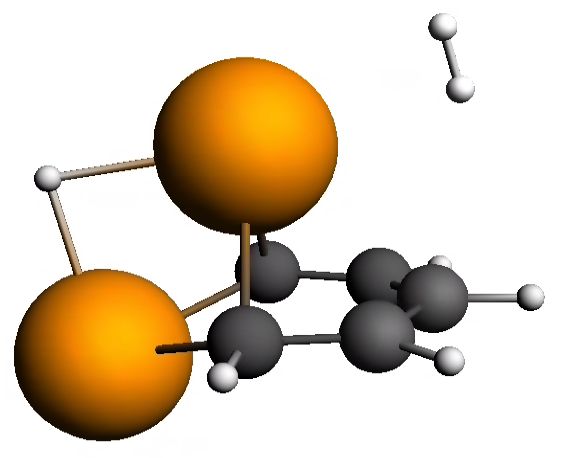 |  | 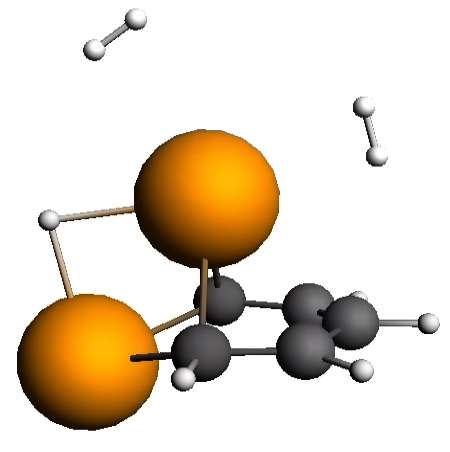 |
|  | 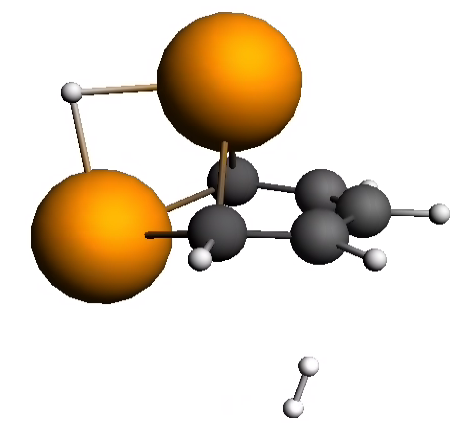 |  | 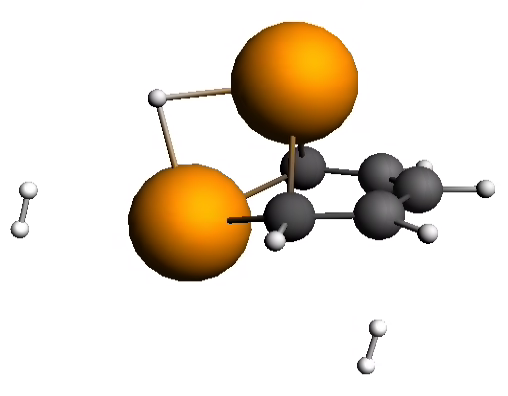 |
| C_5_H_6_BeMg | 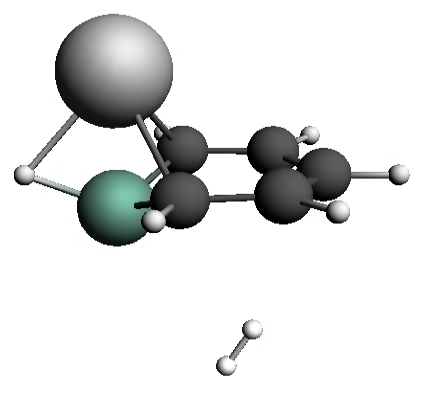 |  | 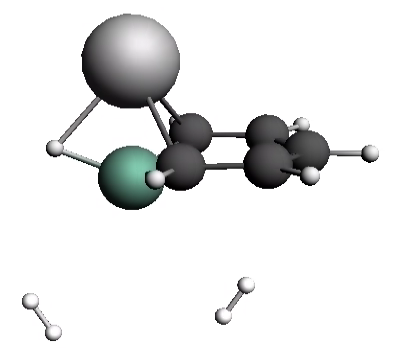 |
|  | 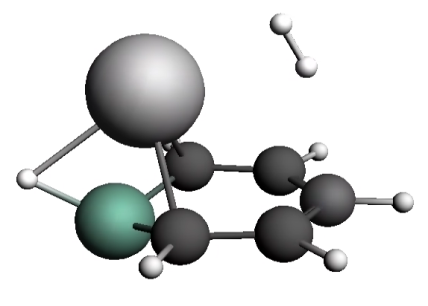 |  | 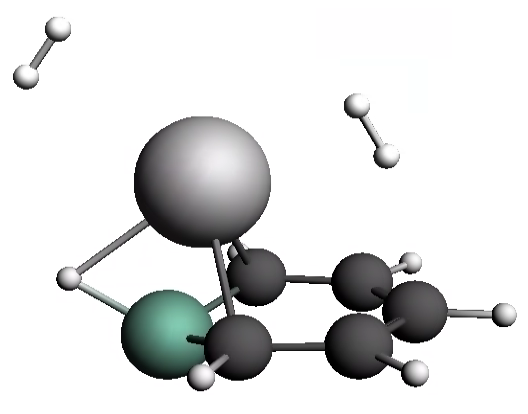 |
| C_5_H_6_BeCa | 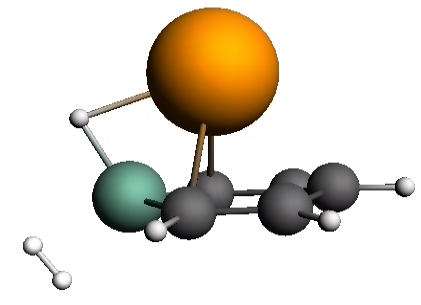 |  | 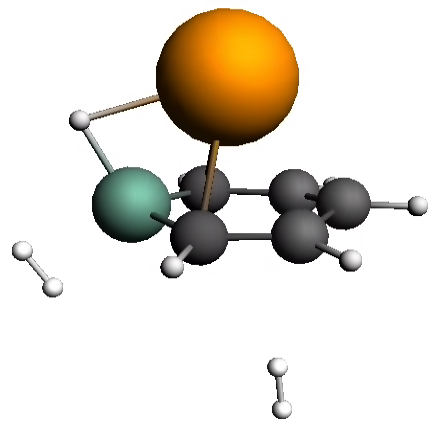 |
|  | 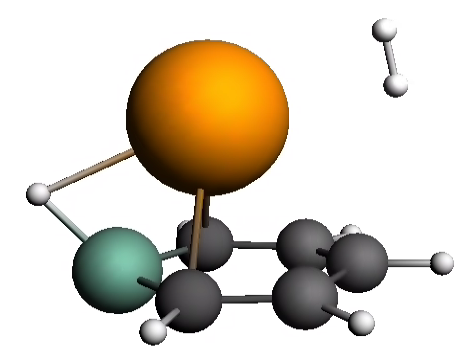 |  | 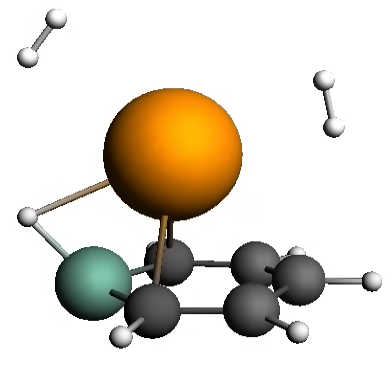 |
| C_5_H_6_MgCa | 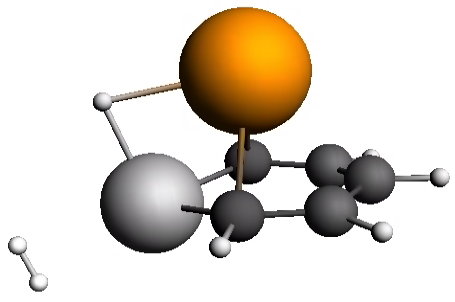 |  | 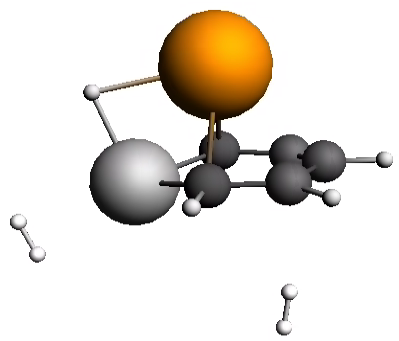 |
|  | 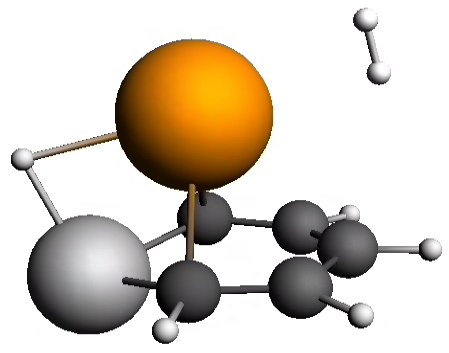 |  | 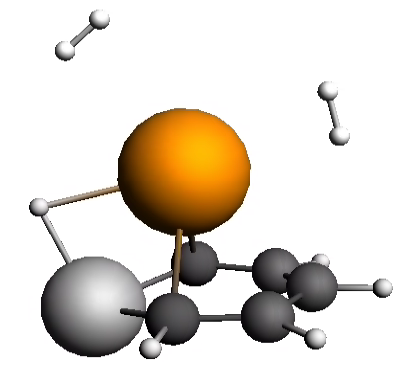 |
